# Ingested metallic foreign body impacted in the vermiform appendix presenting as acute appendicitis: Case report

**DOI:** 10.1016/j.ijscr.2019.03.052

**Published:** 2019-04-05

**Authors:** Ayad Ahmad Mohammed, Dezhwar Yahya Ghazi, Sardar Hassan Arif

**Affiliations:** a University of Duhok, College of Medicine, Department of Surgery, Duhok city, Kurdistan Region, Iraq; b Duhok Directorate of Health, Duhok Emergency Teaching Hospital, Duhok city, Kurdistan Region, Iraq

**Keywords:** Appendicitis, Appendicectomy, Foreign body appendicitis, Vermiform appendix

## Abstract

**Introduction:**

Ingestion of foreign bodies and their impaction at the lumen of the appendix is a very rare finding and only few cases have been reported all over the world. A variety of metallic foreign bodies when ingested may be lodged in the lumen of the appendix such as screws, bird shots, and needles. This is called in most literatures as foreign body appendicitis.

**Case presentation:**

A 4-year-old boy with history of an accidental ingestion of a metallic nail presented to the emergency department one week later with right iliac fossa pain and one attack of vomiting. During abdominal examination there was tenderness and rebound tenderness at the right iliac fossa. Plain abdominal X-ray showed the metallic nail in the region of the right iliac fossa. Ultrasound examination was normal apart from tenderness of putting the probe on the right lilac fossa. The white blood cell counts were 14,000 cell per microliter.

During surgery the nail was found to be impacted inside the lumen of the vermiform appendix causing inflammation of the appendix. Appendicectomy done and the patient discharged on the third day in a good general condition.

**Conclusion:**

Acute appendicitis may be caused by a variety of causes including ingested foreign bodies if impacted in its lumen. When the patient has signs of generalized peritonitis it is important to exclude bowel perforation. The surgery can be done by the open surgery or laparoscopically.

## Highlights

• Foreign body appendicitis in children may be caused by a variety of ingested foreign bodies.
• Pins are the most common cause of foreign body appendicitis.
• The reported incidence of bowel perforation is less than 1%, especially with sharp, thin, pointed or long objects.

## 1 Introduction

Acute appendicitis is one of the commonest surgical causes that present to the emergency department with abdominal pain. Most patients present with the classical signs of appendicitis; i.e. peri-umbilical pain that shifts to the right iliac fossa, anorexia, nausea and vomiting. When these symptoms are present, it makes the diagnosis of appendicitis the most probable cause but investigations are needed to exclude other differential diagnoses [1].

During examination patients may have tenderness and guarding at the region of the right iliac fossa, rebound tenderness, elevated temperature and pulse rate. Investigations that are done in suspected cases of acute appendicitis may include the white blood cell count which shows leukocytosis and shift to the left of neutrophils, elevated inflammatory markers such as the C-reactive protein, ultrasound examination and CT-scan are very important in diagnosing or excluding acute appendicitis in most of the patients [[1], [2], [3], [4], [5]].

Graded compression of the right iliac fossa by the ultrasound probe during sonography is an effective way in eliciting the degree of tenderness and the diagnosis of acute appendicitis [6].

The management of acute appendicitis is mostly through surgical removal of the vermiform appendix either through open surgery or laparoscopically. Treatment with antibiotics alone may be done in non-complicated group of patients [7].

The work of this case report has been reported in line with the SCARE criteria [8].

## 2 Patient information

A 4-year-old boy with history of an accidental ingestion of a metallic nail presented to the emergency department few hours after the ingestion and the examination was normal at that time, the plain abdominal X-ray confirmed the presence nail in the abdomen. The parents were reassured about the possibility of the spontaneous passage of the nail with the stool and they have been advised to visit the hospital if the child develops any kind of symptom. One week later he presented with right iliac fossa pain and one attack of vomiting.

### 2.1 Clinical findings

During abdominal examination, the abdomen was not distended and soft, there was tenderness and rebound tenderness at the right iliac fossa, the bowel sounds were normal and no palpable mass detected.

### 2.2 Diagnostic assessment

**Fig. 1:**
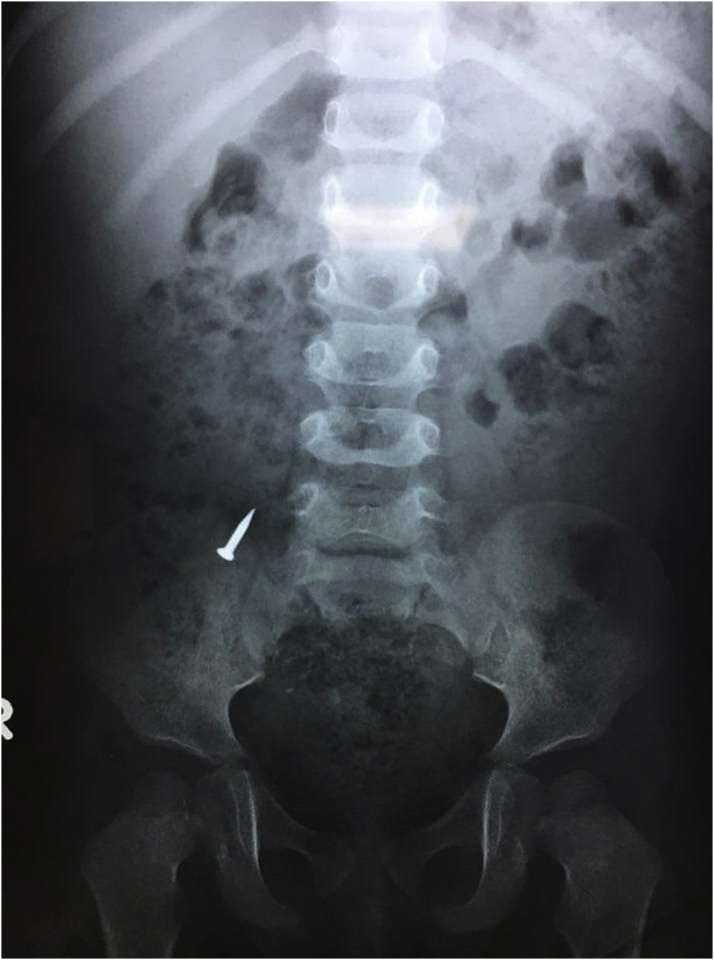
Plain abdominal X-ray in erect position showing the metallic nail in the region of the right iliac fossa.

Plain abdominal X-ray of the abdomen showed the metallic nail in the region of the right iliac fossa, with no other abnormal finding, Fig. 1. Ultrasound examination of the abdomen showed no fluid collection and there was tenderness on putting the probe on the region of the right lilac fossa.

The white blood cell count was elevated (14,000 cells per microliter).

### 2.3 Therapeutic intervention

**Fig. 2:**
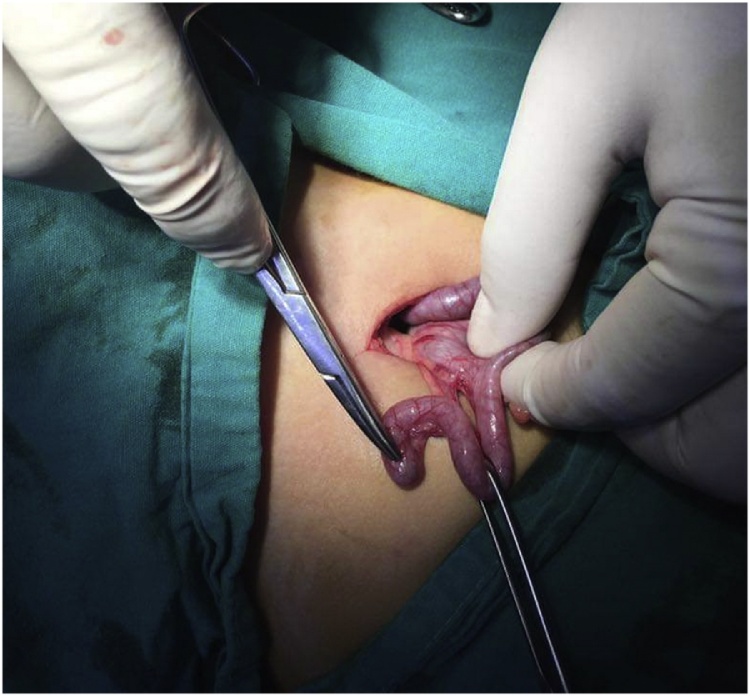
An intraoperative picture showing the metallic nail impacted at the tip of the vermiform appendix.

**Fig. 3:**
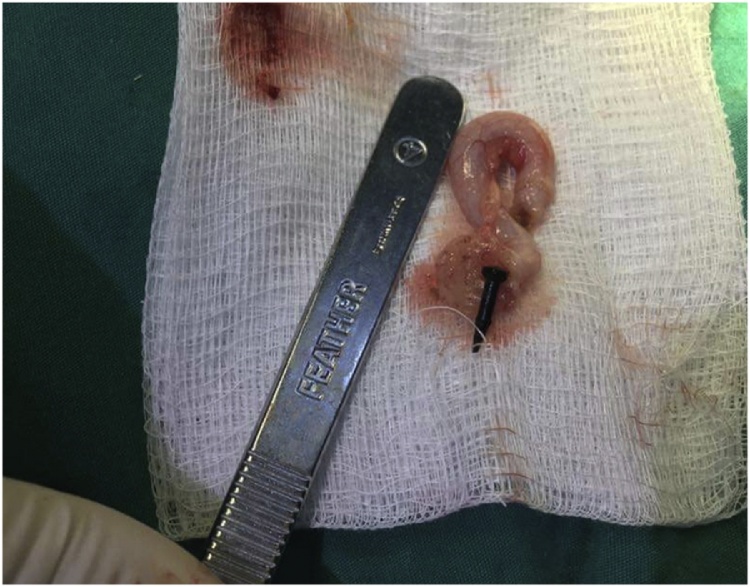
Showing the metallic nail extracted after the tip of the vermiform appendix has been opened after appendicectomy.

Before surgery we suspected impaction at the ileocecal junction and if surgery is delayed it may lead to bowel perforation. During surgery surprisingly the nail was impacted the lumen of the vermiform appendix causing inflammation Fig. 2, Fig. 3.

Appendicectomy done and the patient admitted for two days with no postoperative complications.

### 2.4 Follow-up and outcomes

The patient discharged on the third day in a good general condition.

## 3 Discussion

Acute appendicitis that is caused by an impacted foreign body inside its lumen is an extremely rare finding, the lumen of the appendix may be obstructed with concretions called appendicolithiasis or fecaliths which may be detected in up to 10% of patients that are operated for acute appendicitis, other causes of luminal obstruction may include parasites like *Enterobius vermicularis, Entamoeba histolytica*, and schistosomal appendicitis which occur in very rare occasions. Infection with *Enterobius vermicularis* is considered the most common helminthic infection affecting the gastrointestinal tract worldwide, this infection tends to occur in all ages but it has certain predilection to the children and the young people. The adult worm lives in the terminal ileum and the cecum, most infected patients are asymptomatic but it may cause a variety of manifestations including impaction in the lumen of the appendix causing luminal obstruction. After appendectomy histopathological examination showed that up to 4% of the samples contain the intraluminal worms. The presence of these parasites may initiate the inflammatory process inside the wall of the vermiform appendix together with luminal obstruction. In schistosomal appendicitis there may also be peri-appendicular inflammatory process against the parasite leading to chronic inflammatory stricture and luminal obstruction. This obstruction of the lumen may result in impaired blood supply and progression of the inflammatory process [[9], [10], [11]].

Impaction of ingested foreign bodies inside the lumen of the appendix is a very rare finding and only few cases have been reported all over the world. This condition is called foreign body appendicitis. A variety of metallic foreign bodies when ingested may be lodged in the lumen of the appendix such as screws, bird shots, and needles. The incidence of bowel perforations is rare but may occur in about 1% of patients specially with sharp ended objects. The management of such cases if diagnosed preoperatively is by surgical removal of the appendix whether by the open or laparoscopic technique with no specific preoperative considerations [12,13].

### 3.1 Patient perspective

The family was happy because the metallic nail was extracted and the appendix have been removed in a single surgery.

## Conflicts of interest

The authors have no conflicts of interest to declare.

## Sources of funding

None.

## Ethical approval

Ethical approval has been exempted by my institution for reporting this case.

## Consent

An informed consent is taken from the parents on behalf of the patient for publishing this case report.

## Author contribution

Dr Ayad Ahmad Mohammed contributed to the concept of reporting the case and the patient data recording.

Drafting the work, design, and revision done by Dr Ayad Ahmad Mohammed and Dr Sardar Hassan Arif.

Dr Ayad Ahmad Mohammed and dr Dezhwar Yahya Ghazi took the consent from the patient for publishing the case.

Final approval of the work to be published was done by Dr Sardar Hassan Arif, Dr Ayad Ahmad Mohammed, and Dr Dezhwar Yahya Ghazi.

## Registration of research studies

This work is case report and there is no need of registration.

## Guarantor

Dr Ayad Ahmad Mohammed is guarantor for the work.

## Provenance and peer review

Not commissioned, externally peer-reviewed.
